# Analysis of MiRNA-17 and MiRNA-146 Expression During Differentiation of Spermatogonial Stem Like Cells Derived from Mouse Bone Marrow Mesenchymal Stem Cells

**DOI:** 10.22088/IJMCM.BUMS.8.1.14

**Published:** 2019-07-23

**Authors:** Saba Behzadi Fard, Zohreh Mazaheri, Nasim Ghorbanmehr, Mansoureh Movahedin, Mahin Behzadi Fard, Mohammad Ali Gholampour

**Affiliations:** 1 *Department of Anatomical Sciences, Faculty of Medicine, Tarbiat Modares University, Tehran, Iran.*; 2 *Biotechnology Department, Faculty of Biological Sciences, Alzahra University, Tehran, Iran.*; 3 *Dezful University of Medical Sciences, Dezful, Iran.*; 4 *Hematology Department, Faculty of Medicine, Tarbiat Modares University, Tehran, Iran.*

**Keywords:** Bone marrow mesenchymal stem cells, spermatogonial stem like cells, differentiation, miRNA-17, miRNA-146

## Abstract

*In vitro* derivation of germ cells from different stem cell sources has been challenging in the treatment of male infertility. MicroRNAs (miRNAs) have an essential role in gene expression at post-transcriptional level. The aim of this research was to find more about miRNA-17 and miRNA-146 expression during differentiation of spermatogonial stem cell like cells (SSC like cells) from mouse bone marrow mesenchymal stem cells (BMSCs) through bone morphogenic protein 4 (BMP4) and retinoic acid (RA) induction. BMSCs were treated with BMP4 to produce primordial germ cell like cells (PGC like cells). The cells were differentiated into SSC like cells by an inducer cocktail including RA, leukemia inhibitory factor (LIF) and basic fibroblast growth factor (bFGF). The PGC like cells and SSC like cells were evaluated for pluripotency (*Nanog*, *Oct-4*) and germ cell specific gene (*Piwil2*, *Plzf*, *Dazl*, and *Stra8*) expression, protein expression (Plzf, Stra8), and miRNA-17 and miRNA-146 mRNA expression. Our results showed that BMP4 leads to *Dazl* upregulation and *Nanog* downregulation expression in PGC like cells. RA upregulated *Stra8* and *Piwil2*, and downregulated *Nanog* and *Oct-4*. MiRNA-17 and miRNA-146 expression decreased significantly in SSC like cells after RA treatment. This research indicated the aberrant miRNA-17 and miRNA-146 expression in SSC like cells in comparison with SSCs. Downregulation of the two miRNAs using RA in the stimulated undifferentiated state could probably be one of the key factors of SSC like cell arrest.

Over the years, significant number of investigations has been focused on male infertility problems based on stem cell therapy (1). *In vitro* derivation of germ cells from different sources of stem cells has been studied to solve male infertility (2). Bone marrow mesenchymal stem cells (BMSCs) are known widely as appropriate stem cells that have capacity to self-renew and differentiate into several cell types such as bone, cartilage, fat, muscle, tendon, liver, and perimordial germ cells (PGCs) (3). Numerous *in vitro* reports have supported the ability of mesenchymal stem cells (MSCs) to generate germ cells (4, 5). The recent findings have reported a variety of inducers and culture systems to reach *in vitro* germ cells (6, 7).

It has been proposed that bone morphogenetic protein 4 (BMP4) and retinoic acid (RA) could promote MSCs transdifferentiation into germ cells (8, 9). It is believed that BMP4 signaling has a crucial role in germ cell specification in both *in vivo* and *in vitro* approaches (10). Another critical regulator of male germ cell fate RA is important for both the initiation of differentiation, and the entry into meiosis in male germ cells (11). Despite this impressive body of investigations, *in vitro*-derived or arrest of male germ cell in specific stage which could be due to inefficient cell population, and poorly-defined regulatory mechanisms involved in gene expression control in male germ cell differentiation process.

MicroRNAs (miRNAs) are an important class of non-coding RNAs (ncRNAs) involved in gene expression regulation at post-transcriptional level (12). Mature miRNAS are single-stranded 19-25 nucleotides incorporated into RNA-induced silencing complex, and act through targeting the 3^´^-untranslated region (3´UTR) of mRNAs resulting in translational repression and/or target mRNA degradation (13). The importance of miRNA-mediated translational repression in spermatoge-nesis process has been investigated by deletion of dicer1 as a crucial agent for miRNA biogenesis (14-16). Notably, impaired biogenesis of miRNAs could disrupt spermatogenesis and lead to infertility in male mice (17).

miRNA-17 is a member of Mir-17-92 cluster that modulates pluripotency network (18). Deregulation of this cluster plays a role in cancer progression (19). More recently, Tong et al. have identified miRNA-17-92 cluster as a regulatory factor in germ cell development (20).

The distinct role of miRNA-146 in spermato-genesis was demonstrated by Huszar et al. who showed that miRNA-146 is an important factor in maintaining spermatogonia in an undifferentiated state. Additionally, miRNA-146 is able to directly affect spermatogonial differentiation by modulating RA-associated differentiation markers such as kit oncogene (Kit), stimulated by retinoic acid gene 8 (Stra8), and spermatogenesis- and oogenesis-specific basic helix-loop-helix2 (Sohlh2) (21).

Collectively, little is known about miRNA-17 expression in male germ like cells. In the current study, we used BMP4 and RA cocktail including leukemia inhibitory factor (LIF) and  basic fibroblast growth factor (bFGF) to induce mouse BMSCs into PGC like cells, and then spermatogonial stem cell like cells (SSC like cells). We evaluated miRNA-17 and miRNA-146 mRNA expression changes at each step by real time-PCR.

## Materials and Methods

**Isolation and culture of mouse BMSCs**

Bone marrow aspirated from mouse (NMRI 6-8 weeks) tibia and femur and cultured in Dulbecco’s Modified Eagle’s Medium-F12 (DMEM-F12) (Gibco, Germany) supplemented with 15% FBS (Gibco, UK), 100 U/ml Penicillin/Streptomycin (Gibco, Germany), 0.1 mM β-mercaptoethanol (Sigma, USA), placed in an incubator in humidified atmosphere at 37 °C and 5% CO_2 _in air. BMSCs at confluence of >80% were sub-cultured to reach passage 3.

**Characterization of BMSCs**

***Immunocytochemistry of BMSCs***

Immunocytochemistry was performed to investigate the expression of mesenchymal markers including CD44 and CD105 in passage 3 of BMSCs. The expression of these markers was examined using their related antibodies. To this end, BMSCs were washed with cold PBS, and fixed with 4% cold formaldehyde for 20 min. For permeabilization for 15 min, 0.05% Triton X-100 was used, followed by blocking with 10% goat serum for 45 min at room temperature. After blocking, the cells were incubated at 4 °C overnight with primary antibody against CD44 (1: 100, rabbit polyclonal IgG, Abcam, Cambridge, MA, USA) and CD105 (1:100, rabbit polyclonal IgG, Abcam, Cambridge, MA, USA). After three times washing, the secondary antibodies including goat anti-rabbit IgG conjugated with FITC (1:200, Abcam, Cambridge, MA, USA) were added to the cells, and incubated in the dark at room temperature for 3 h. The cells were then washed twice with PBS for 5 min. The cells were viewed under a fluorescent microscope (Olympus, Shinjuku, Japan) after their nuclei were stained with propidium iodide (PI).

***Induction of osteogenic and adipogenic differen-tiation***

To study the differentiation potential of BMSCs cells, confluent cells at passage 3 were cultured in differentiation media for osteogenic and adipogenic induction. Adipogenic differentiation medium was consisted of DMEM containing 10% FBS and 100 nM dexamethasone. Osteogenic differentiation medium consisted of DMEM containing, 10 nM β-glycerophosphate, 80 µg/ml ascorbic acid and 10 nM dexamethasone.The medium was changed every 4 days. After twenty-one days, the cultures were used for histochemical staining such as oil red and alizarin red S (all Sigma, St. Louis, USA).

***Induction of PGC like cell differentiation***

For PGC differentiation, confluent BMSCs at passage 3 were cultured at concentration of 1×10^6^ cells in 25 cm^2^ culture flasks at 37 °C in humidified atmosphere with 5% CO_2_ in differentiation medium containing 25 ng/ml BMP4

for 4 days.

***Induction of SSC like cell differentiation***

After BMP4 treatment, RA (10^-5^ M), LIF (1000 U/ml) and bFGF (1 ng/ml) were added for a culture period of 14 days. The medium was changed every 4 days.

**Immunocytochemistry of** ***in vitro*** **treated cells**

Immunocytochemistry was performed as described previously to evaluate the expression of Plzf and Stra8 proteins in PGC like cells and SSC like cells, respectively. Primary antibodies, rabbit polyclonal IgG, and secondary goat anti-rabbit IgG conjugated with FITC (Abcam, Cambridge, MA, USA) were used.

**Table 1:** Primers used for Real time PCR

Genes	Primer sequence (5´->3')	Length	T_m_
Β-actin	Forward: TTACTGAGCTGCGTTTTACAC Reverse: ACAAAGCCATGCCAATGTTG	91	77
Nanog	Forward: CGAGGATGAGACAGAAC Reverse:CCAAGGACAAGCAAGCAC	170	77.6
Oct-4	Forward: AGCACGAGTGGAAAGCAAC Reverse: AGATGGTGGTCTGGCTGAAC	210	78.2
Piwil2	Forward: CCTCCAGCTCTGTCTCCAAC Reverse: CCTTGCTTGACCAAAAGCTC	95	77.6
Plzf	Forward:CGGAGAGGAACCTGAAGC Reverse:CGCCAATATCTGATGAAGC	161	75
Dazl	Forward: AAGGCAAAATCATGCCAAAC Reverse: TCCTGATTTCGGTTTCATCC	72	78.72
Stra8	Forward: CTCCTCCTCCACTCTGTTGC Reverse: GCGGCAGAGACAATAGGAAG	135	78.5

**Fig. 1.**
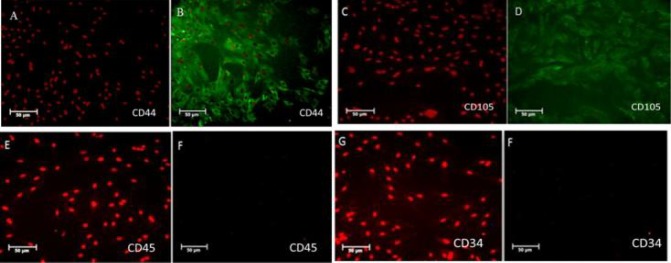
Characterization of BMSCs. A-D: CD44, CD105 as mesenchymal stem cell surface markers; E-H: CD45, CD34 as hematopoietic cell surface markers

**Fig. 2:**
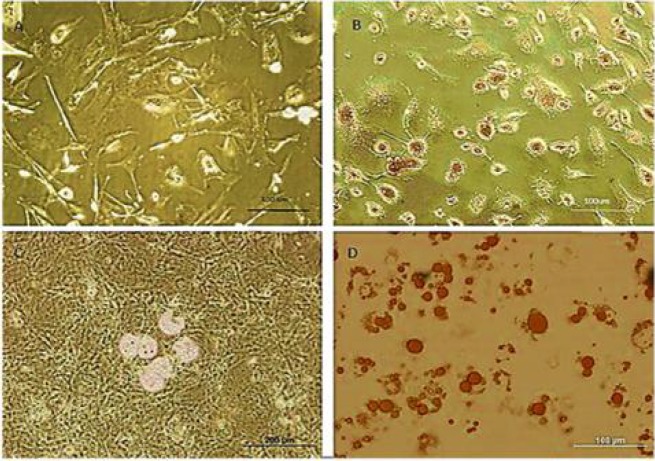
BMSCs differentiation to osteogenic and adipogenic lineages. A, B: osteogenic lineage derived from BMSCs (alizarin red-S staining); C, D: adipogenic lineage derived from BMSCs (oil red-O staining)

**Fig. 3:**
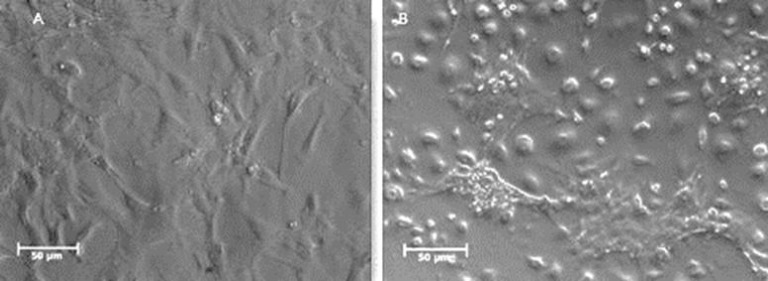
BMSCs morphology after BMP4 treatment. BMSCs morphology before (A) and after BMP4 treatment (B).

**Fig. 4:**
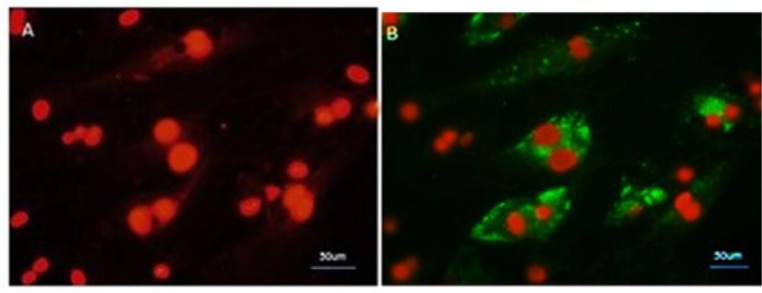
Immunostaining for Plzf protein . BMSCs treated with BMP4 for 4 days. (A) Nuclei were stained with PI. (B) Merged image indicated Plzf-positive cells

**Fig. 5:**
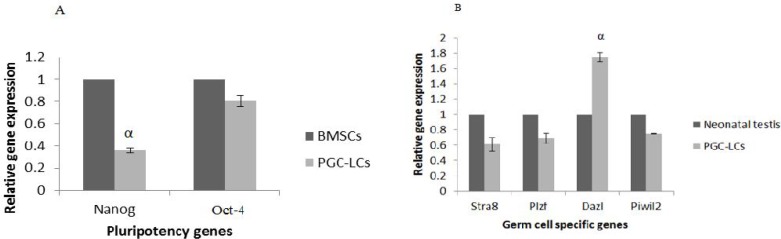
PGC like cells real Time-PCR analysis. mRNA levels were normalized with respect to β-actin, chosen as an internal control. Pluripotency (A) and germ line (B) genes expression of PGC like cells. BMSCs and neonatal testis were chosen as control groups. Histograms show mean expression values (± SD, n=3; P < 0.05).

**Fig. 6:**
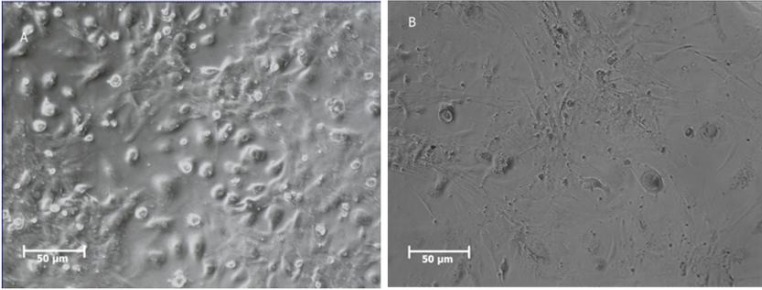
morphology of PGC like cells after RA treatment. Morphology PGC like cells before (A) and after (B) RA treatment

**Fig. 7:**
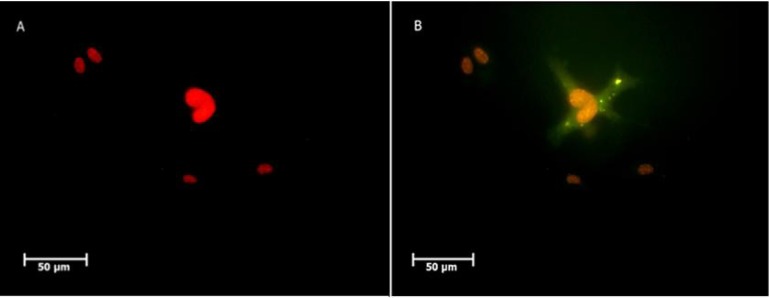
Immunostaining for Stra8 protein. PGC like cells treated with RA for 14 days. A: before treatment; B: After treatment. Nuclei were stained with PI

**Fig. 8:**
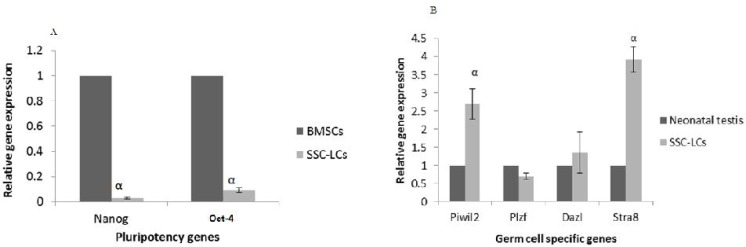
SSC like cells real Time-PCR analysis. mRNA levels were normalized with respect to β-actin, chosen as an internal control. Pluripotency (A) and germ line (B) gene expression of SSC like cells. BMSCs and neonatal testis were chosen as control groups. Histograms show mean expression values (± SD, n=3; P < 0.05)

**Fig. 9:**
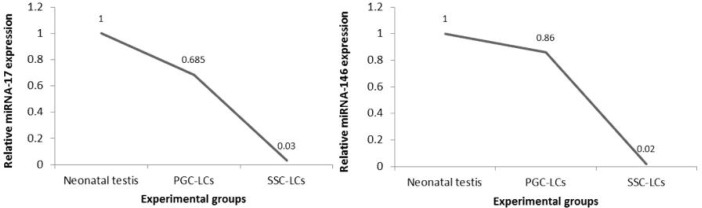
Selected miRNAs real time PCR analysis. MiRNA-17 (A) and miRNA-146 (B) expression of PGC like cells and SSC like cells. MiRNA levels were normalized with respect to U6, chosen as an internal control. Neonatal testis was chosen as control group. Histograms show mean expression values (± SD, n=3; P < 0.05)

**RNA isolation and real time PCR**

Total RNA was isolated from treated cells and BMSCs and neonatal testis as controls using TRIZOL isolation reagent. cDNAs for pluripotency and germ specific mRNAs were synthesized with a BioFact™ RT Series (Biofact Co, Korea) using oligo (dT) primers (1 µl), 2X RT Pre-Mix (10 µl) , extracted RNA (1 µg) and RNase-free water. *Nanog*, *Oct4*, *Piwil2*, *Plzf*, *Dazl*, *Stra8*, and β actin (as internal control) primers are presented in table 1. The cDNAs of miRNA-17 and miRNA-146 were synthesized using miScript II RT Kit (Qiagen, USA). The real time PCR was performed according to the manufacturer’s recommendations (Applied Biosystems, Foster City, CA, USA). Each PCR reaction contained 1000 ng cDNA, 10 µl SYBR-Green, 0.5 µl of predesigned primers in a total volume of 20 µl. The cycles were 50 °C for 2 min, 95 °C for 10 min, followed by 45 cycles of 95 °C for 15 s and 60 °C for 1 min.

**Statistical analysis**

Statistical analysis was performed using SPSS (v.19) and student’s t-test. One-way ANOVA and Tukey post-hoc tests were used to determine the statistical significance among groups. The data are presented as mean±SD (P <0.05).

## Results

**BMSCs culture and cell markers expression**

The isolated BMSCs after 3 passages demonstrated a fibroblast-like phenotype. Immuno-cytochemistry analysis showed that the cells expressed CD44 (96%) and CD105 (95.4%), and did not express hematopoietic cells surface markers CD34 and CD45 (8.36% and 6.45%, respectively) (Figure 1). The differentiation potential of BMSCs was achieved by culturing the cells for 21 days in differentiation media. Adipogenic differentiation was approved by oil red O-positive cells, and osteogenic differentiation, by alizarin red S-positive cells (Figure 2).

***In vitro*** **PGC like cells differentiation from BMSCs**

To achieve PGC like cells, BMSCs were treated with BMP4 for 4 days and characterized by morphological changes, Plzf immunostaining and real time PCR. After treatment, spindle -like BMSCs showed round shape and cluster accumulation (Figure 3). 

Immunocytochemistry test showed that 51.7% of BMP4 treated cells were positive for Plzf protein (Figure 4).

Real time analysis showed that *Nanog* expression decreased significantly (P≤0.05) after BMP4 treatment in PGC like cells in comparison with BMSCs non-treated cells as control group. Also, *Dazl* expression increased significantly (P ≤0.05) in PGC like cells after BMP4 treatment in comparison with neonatal testis cells as control group (Figure 5).

***In vitro*** **SSC like cells differentiation from BMSCs-derived PGC like cells**

As described previously, BMP4 treatment was followed by RA cocktail for 14 days to induce SSC like cell differentiation. After this culture period RA treated cells showed morphological changes as shown in Figure 6.

Immunocytochemistry test showed that 54.06% of RA treated cells were positive for Stra8 protein as a specific marker after RA induction (Figure 7).

Real time analysis showed that *Nanog* and *Oct4* expression decreased significantly (P ≤ 0.05) after RA treatment in SSC like cells in comparison with non-treated BMSCs as control group. Also, *Piwil2* and *Stra8* expression increased significantly (P≤0.05) in SSC like cells after RA treatment in comparison with neonatal testis cells as control group (Figure 8).

**miRNA-17 and miRNA-146 expression in PGC like cells and SSC like cells**

Real Time-PCR analysis demonstrated that miRNA-17 and miRNA-146 expression did not change significantly after BMP4 treatment in PGC like cells. But after RA treatment, both miRNA-17 and miRNA-146 expression level decreased significantly (P ≤0.05) in SSC like cells (Figure 9).

## Discussion

*In vitro* derivation of SSC like cells has been reported in multiple investigations (22, 23). In this study, we used a protocol based on Mazaheri et al.’s investigation to reach SSC like cells (24). According to this approach, after BMP4 treatment, *Nanog* expression decreased significantly in primordial germ cell like cells (PGC like cells) and SSC like cells. *Nanog* is required for maintaining the cells pluripotency (25). Also, knockout experiments show that *Nanog* has a key role in maintaining the proliferation of PGCs (26). Downregulation of nanog might occurr via *Dazl* upregulation. In our study, BMP4 treatment caused *Dazl* over-expression in comparison with non-treated BMSCs. Dazl is a germ cell-specific RNA binding protein (27). Loss of *Dazl *is associated with germ cells failure to be mature during spermatogenesis and induced apoptosis in PGCs (28).

Our experiments showed that using inducer cocktail with RA resulted in PGC like cell differentiation into SSC like cells with significant decrease in pluripotency gene expression that are essential to survival in differentiated cells. Conversely, Piwil2 and Stra8 expression increased after RA treatment. Previous studies reported that Piwil2 (Miligene) has critical functions in spermatogonial stem cell self-renewal, and also modulates expression of Stra8 (29). *Stra8* plays an essential role in germ cell differentiation and can be regulated by RA (30).

The importance of the miRNAs for directing the expression of genes essential for spermatogenesis has been highlighted by several studies (31). Male germ cell-specific miR-17-92 knockout in mice led to mild defect in spermatogenesis (32). Tong et al. found that highly expressed miR-17-92 in undifferentiated SSCs were significantly down-regulated after RA-induced spermatogonial differentiation. Using miRNAs array and bioinformatic analyses revealed *Bcl2l11*, *Kit*, *Socs3*, and as validated targets of the Mir-17-92 cluster upregulated upon RA- induced spermatogonial differentiation (20). In our study, we showed downregulation of miRNA-17 as a member of Mir-17-92 cluster during *in vitro* RA-induced spermatogonial differentiation.

Spermatogonial stem cells as one of the undifferentiated spermatogonia cells are capable of both self-renewability and differentiation to promote spermatogenesis (22). Huszar et al. demonstrated that miRNA-146 expression level was significantly upregulated in undifferentiated spermatogonia cells in *in vivo* model. Additionally, miRNA-146 expression level decreased in differentiated spermatogonia by RA treatment (21).

Overall, our research indicated that following RA exposure, miRNA-146 and miRNA-17 expression were significantly downregulated in SSC like cells differentiated from PGC like cells that are considered having stem cell potential. These data support our hypothesis inducing differentiation-associated genes based on evidence whch is not possibly sufficient to promote *in vitro* cell fate transition. We concluded that likely the two miRNAs downregulation using RA in stimulated undifferentiated state may be probably one of the key factors of SSC like cell arrest. The exact relationship between the two miRNAs expression and *in vitro* germ cell differentiation promotion needs to be further explored in the future.

## Conflict of interests

The authors declare that there is no conflict of interest.

## Funding

This work was supported by grants from Medical Faculty of Tarbiat Modares University, Tehran, Iran.
